# Performance of combined persulfate/aluminum sulfate for landfill leachate treatment

**DOI:** 10.1016/j.dib.2018.05.111

**Published:** 2018-05-24

**Authors:** Salem S. Abu Amr, Abbas F.M. Alkarkhi, Tamer M. Alslaibi, Mohammed Shadi S. Abujazar

**Affiliations:** a MICET),Melaka,Malaysian Institute of Chemical and Bioengineering Technology, Universiti Kuala Lumpur (UniKl, MICET), Alor Gajah 78000, Melaka, Malaysia; b Civil Engineering Department, Palestine University, Gaza Strip, Palestine; c Faculty of sciences, Al-Aqsa University, Gaza, Palestine

**Keywords:** Oxidation, Persulfate, Activation, Leachate treatment, Optimization

## Abstract

Although landfilling is still the most suitable method for solid waste disposal, generation of large quantity of leachate is still considered as one of the main environmental problem. Efficient treatment of leachate is required prior to final discharge. Persulfate (S_2_O_8_^2−^) recently used for leachate oxidation, the oxidation potential of persulfate can be improved by activate and initiate sulfate radical. The current data aimed to evaluate the performance of utilizing Al_2_SO4 reagent for activation of persulfate to treat landfill leachate. The data on chemical oxygen demand (COD), color, and NH_3_–H removals at different setting of the persulfate, Al_2_SO_4_ dosages, pH, and reaction time were collected using a central composite design (CCD) were measured to identify the optimum operating conditions. A total of 30 experiments were performed, the optimum conditions for S_2_O_8_^2−^/Al_2_SO_4_ oxidation process was obtained. Quadratic models for chemical oxygen demand (COD), color, and NH_3_–H removals were significant with p-value < 0.0001. The experimental results were in agreement with the optimum results for COD and NH_3_–N removal rates to be 67%, 81%, and 48%, respectively). The results obtained in leachate treatment were compared with those from other treatment processes, such as S_2_O_8_^2−^ only and Al_2_SO_4_ only, to evaluate its effectiveness. The combined method (i.e., /S_2_O_8_^2−^/Al_2_SO_4_) showed higher removal efficiency for COD, color, and NH_3_–N compared with other studied applications.

**Specifications Table**

**Table:** 

**Subject area**	Environmental Engineering
**More specific subject area**	Landfill leachate treatment
**Type of data**	Equations and statistical data
**How data was acquired**	All experiments performed in 250 mL glass conical flasks orbital shaker unit, Aluminium sulphate (ZnSO_4_) was used to initiate sulphate radicals from persulfate (Na_2_S_2_O_8_) and improve the oxidation potential. The concentration of COD, color, and ammonia was measured before and after each run and the removal efficiencies were calculated.
**Data format**	Table, Figure, Equation
**Experimental factor**	Monitoring the removal efficiencies of COD, colour, COD, and ammonia from leachate.
**Experimental features**	Response surface methodology (RSM) was used to design the experimental conditions for treatment of leachate using simultaneous persulfate/ZnSO_4_ oxidation. Removal efficiency of COD, colour, and ammonia was measured. The relationship between experimental factors (S_2_O_8_/AlSO_4_ ratio dosage, pH and reaction time) and responses (COD, colour, COD, and ammonia) was evaluated.
**Data source location**	Malaysian Institute of chemical & Bioengineering Technology
	Universiti Kuala Lumpur, (UniKL, MICET), 78000, Melaka, Malaysia.
**Data accessibility**	Data are presented in the article

**Value of the data**

• This article presents data on the performance of utilize Al_2_SO_4_ to activate persulfate for leachate treatment.
• The data provides comparison of the treatment efficiencies between persulfate alone, Al_2_SO_4_ alone and combined persulfate/Al_2_SO_4_.
• The data show the relationship between experimental factors and the responses statistically using mathematical models for COD colour and ammonia.
• The optimum results can be useful for wide application on wastewater treatment.

## 1 Data

**Fig. 1:**
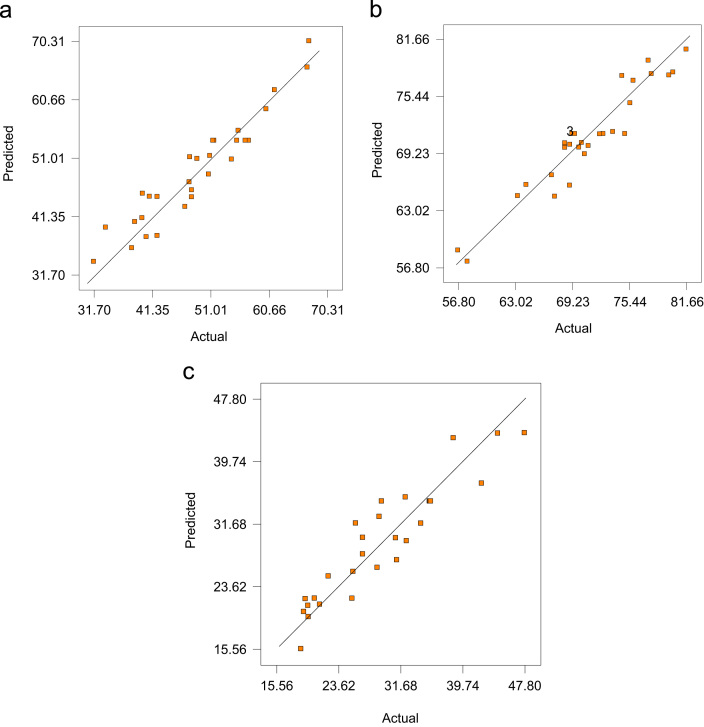
Design expert plot; predicted and actual standardized residual for (A) COD, (B) color (C) NH_3_–N, removal.

**Fig. 2:**
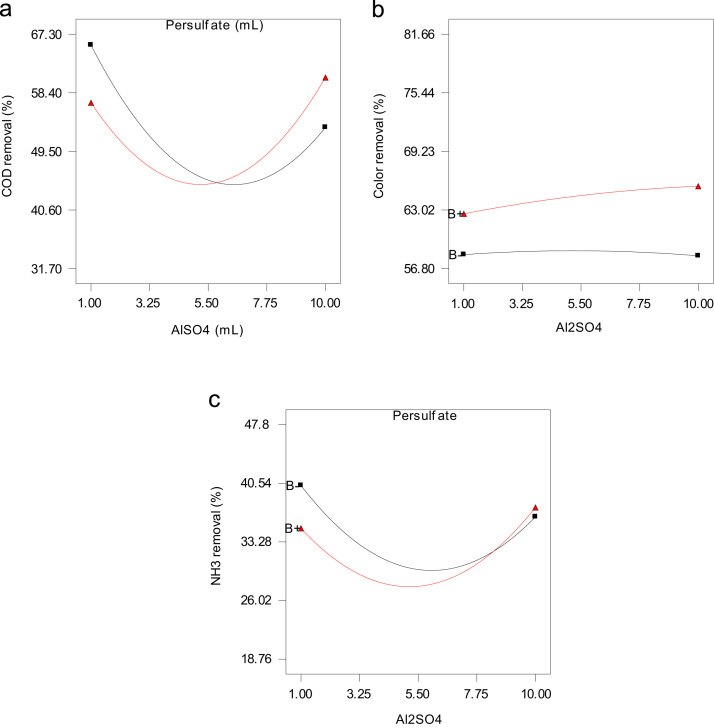
Two-factor interaction plot showing the behavior of Al_2_SO4 and persulfate (■ = 1, ▲= 10 mL) on (A) COD color (B) and (C) NH_3_–N removal at 6 pH and 105 min reaction time.

**Fig. 3:**
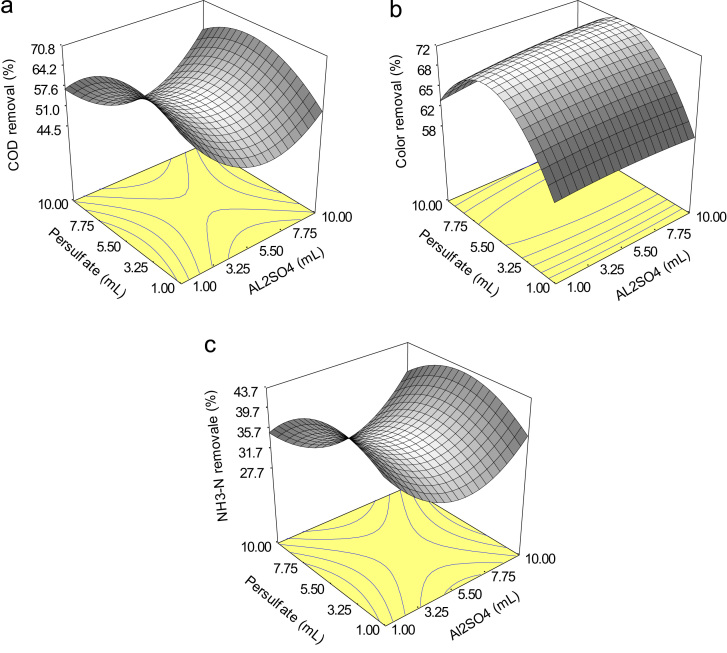
Three-dimensional response surface showing the effect of persulfate/Al_2_SO_4_ on (A) COD (B) color (C) and NH_3_–N removal at 5.5 ml of persulfate and 105 min reaction time.

**Fig. 4:**
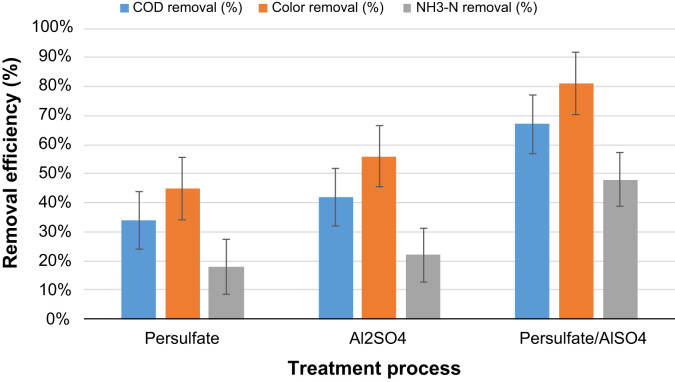
Comparison the performance of persulfate, Al_2_SO_4_ and combined persulfate/Al_2_SO_4_ for COD , color and NH_3_–N removal.

The data for general characteristics of landfill leachate used in this study are presented in (Table 1). Furthermore, the data in this article covers the performance of combined persulfate/aluminum sulfate for leachate treatment based on three measured responses COD, color, and NH_3_-H removals at different setting of the persulfate, Al_2_SO_4_ dosages, pH, and reaction time (Table 2). The data for COD, color, and NH_3_ removals obtained from faced central composite design (FCCD) are presented in Table 3. The significance of the influential variables are presented in Table 4 (analysis of variance (ANOVA)). Mathematical models that show the effect of significant variables on COD, color, and NH_3_–N removals are presented in Eqs. (1), (2), (3) respectively. Fig. 1 shows the predicted and actual standardized residual for COD, color and NH_3_–N, removal. Fig. 2, presents the two-factor interaction plot for the behavior of combined Al_2_SO4 and persulfate on COD color and NH_3_–N removal. Fig. 3. Shows the three-dimensional response surface for the effect of combined Al_2_SO4 and persulfate on COD color and NH_3_-N removal. Fig. 4, compared the treatment efficiency between the three related treatment processes; persulfate, Al_2_SO_4_ and combined persulfate/Al_2_SO_4_ for COD, color and NH_3_–N removal.

**Table 1:** Characteristics of Sungai Udang landfill leachate.

**Parameters**	**Value**^a^
COD (mg/L)	2300
BOD (mg/L)	110
NH_3_–N (mg/L)	870
Color (PT Co.)	4800
pH	8.6
Suspended solids (mg/L)	88
Conductivity, (μS/cm)	18,940

a Average of two samples taken from March and June 2017.

**Table 2:** Independent variables (factors) and corresponding levels used for optimization.

**Variables**	**Symbol**	**Range and levels**		
		Low level (− 1)	Center (0)	**High level + 1**
**Persulfate dosage**	$X_{1}$	1 ml	5.5 ml	**10 ml**
**Al_2_(SO_4_)_3_ dosage**	$X_{2}$	1 ml	5.5 ml	**10 ml**
**pH**	$X_{3}$	3	6	**9**
**Reaction time**	$X_{4}$	**30**	**105**	**180**

**Table 3:** The results of FFCD including coded and actual variable with the results of three responses (Color, COD, NH_3_ removals).

Coded variable				Actual variable				Responses		
Al_2_SO_4_	Persulfate	pH	RT	Al_2_SO_4_	Persulfate	pH	RT	Color removal	COD removal	NH-N removal
− 1	0	0	0	1	5.5	6	105	69	67.3	47.8
0	0	0	0	5.5	5.5	6	105	69.53	51.4	35.6
− 1	1	− 1	− 1	1	10	3	30	70.6	47.9	34.33
− 1	− 1	1	− 1	1	1	9	30	64.24	54.5	31.2
0	− 1	0	0	5.5	1	6	105	56.8	39.78	31.07
0	0	0	− 1	5.5	5.5	6	30	72.24	55.6	26.78
1	1	− 1	− 1	10	10	3	30	77.53	61.6	44.33
1	− 1	− 1	− 1	10	1	3	30	63.31	50.9	42.22
0	0	0	1	5.5	5.5	6	180	77.87	48.8	25.4
1	0	0	0	10	5.5	6	105	73.67	67	38.56
− 1	− 1	− 1	1	1	1	3	180	71	50.7	28.67
1	− 1	− 1	1	10	1	3	180	69.97	38	19.13
− 1	1	1	1	1	10	9	180	75.54	47.5	19.33
− 1	1	− 1	1	1	10	3	180	74.67	38.5	21.2
1	− 1	1	1	10	1	9	180	68.45	31.7	19.73
0	1	0	0	5.5	10	6	105	67.34	47.89	26.78
1	1	− 1	1	10	10	3	180	81.66	40.92	20.53
1	1	1	1	10	10	9	180	70.27	42.2	18.76
− 1	1	1	− 1	1	10	9	30	67.02	39.7	19.67
0	0	0	0	5.5	5.5	6	105	69.53	56.76	35.57
0	0	1	0	5.5	5.5	9	105	75.89	33.67	32.47
0	0	− 1	0	5.5	5.5	3	105	80.22	46.8	32.33
1	1	1	− 1	10	10	9	30	68.45	42.2	25.53
0	0	0	0	5.5	5.5	6	105	69.23	55.37	35.56
− 1	− 1	− 1	− 1	1	1	3	30	57.83	47.6	25.87
1	− 1	1	− 1	10	1	9	30	68.98	40.4	22.33
0	0	0	0	5.5	5.5	6	105	69.53	57.32	35.55
0	0	0	0	5.5	5.5	6	105	74.98	56.8	35.45
− 1	− 1	1	1	1	1	9	180	79.78	60.2	28.93
0	0	0	0	5.5	5.5	6	105	72.62	51.6	29.23

The second-order polynomial model for COD, color, and NH_3_–N removals are given in Eqs. (1), (2), (3), respectively.

(1) $$C\mathrm{OD}=53.88-2.17X_{1}-0.30X_{2}-1.71X_{3}-2.33X_{4}+14.27X_{1}2-9.04X_{2}2-12.64X_{3}2-0.68X_{4}2+4.08X_{1}X_{2}-3.26X_{1}X_{3}-3.09X_{1}X_{4}-1.06X_{2}X_{3}-0.59X_{2}X_{4}+2.79X_{3}X_{4}$$

(2) $$\mathrm{Color}=71.37+0.69X_{1}+2.93X_{2}-0.45X_{3}+3.28X_{4}-0.40X_{1}2-9.77X_{2}2+6.22X_{3}2+3.22X_{4}2+0.76X_{1}X_{2}-1.80X_{1}X_{3}-1.83X_{1}X_{4}-2.66X_{2}X_{3}-1.02X_{2}X_{4}-0.17X_{3}X_{4}$$

(3) $$NH3-N=34.60-0.33X_{1}-1.04X_{2}-2.81X_{3}-3.92X_{4}+8.47X_{1}2-5.78X_{2}-2.31X_{3}2-8.62X_{4}2+1.62X_{1}X_{2}-\;1.81X_{1}X_{3}-2.71X_{1}X_{4}-1.46X_{2}X_{3}-1.18X_{2}X_{4}+2.83X_{3}X_{4}$$

where $Y_{1}$, $Y_{2}$, and $Y_{3}$ represent the COD rem oval, Color removal and ammonia (NH_3_), respectively.

## 2 Experimental design, materials and methods

### 2.1 Leachate Sampling and Characteristics

Leachate samples were collected from the detention pond at Sungai Udang Landfill Site (SULS), Melaka, Malaysia. SULS has an area of 7 ha, receiving approximately 1200 t of municipal solid waste daily and start receiving waste at 1st of April 2015. In this study, the leachate samples were collected 6 times manually from February 2017 to Jun 2017 using 2 L plastic containers. The collected samples were immediately transported to the laboratory, characterized, and stored in cool room to 4 °C. The general characteristics of the leachate used in the study are presented in Table 1. All samples were collected, preserved and analysed by following Standard Methods for the Examination of Water and Wastewater [1].

### 2.2 Experimental Procedures

In the current study, Sodium persulfate (Na_2_S_2_O_8_ M = 238 g/mol) and Aluminum sulfate (Al_2_SO_4_ 342.15 g/mol) were used for advanced oxidation during the oxidation of leachate samples. Several dosages of S_2_O_8_ and Al_2_SO_4_ were gradually mixed with 100 mL of leachate samples to determine the optimum S_2_O_8_^2−^ and Al_2_SO_4_ dosage according to the efficiencies of COD, Color and NH_3_–N removal. Orbital Shaker (Luckham R100/TW Rotatable Shaker 340 mm × 245 mm) with at 200 rpm was used for samples shaking [2]. All experiments were performed at room temperature (28 0 C) using 100 mL leachate samples in conical flasks with a 250 mL capacity. pH of the samples was controlled by using 3 M sulphuric acid solution and 3 M sodium hydroxide solution [3]. All experiments were performed at laboratory of Malaysian Institute of chemical & Bioengineering Technology, University of Kuala Lumpur, Melaka, Malaysia.

### 2.3 Experimental design

The effect of four factors, namely persulfate dosage $(X_{1)}$, Al_2_SO_4_ dosage ($X_{2})$, pH ($X_{3})$ and reaction time $(X_{4})$ on three responses COD ($Y_{1}$), color ($Y_{2}$) and ammonia ( $Y_{3}$) removal efficiencies from leachate was studied. The relationship between the factors and the three responses was modelled and optimized by using face centred composite design (FCCD). FCCD is one of the frequently used design in response surface methodology (RSM) to model and optimize the relationship between the input factors and the output responses. The levels of selected factors were chosen based on literature and preliminary experiments, the actual and coded levels are given in Table 2.

The relationship between the selected factors ($X_{1}$, $X_{2}$, $X_{3}$, $X_{4}$ )and each of the responses ($Y_{1}$, $Y_{2}$, $Y_{3}$).is usually described in response surface methodology (RSM) by a second-order polynomial as given in Eq. (4).

(4) $$Y=\beta_{0}+\sum_{i=1}^{4}\beta_{i}X_{i}+\sum_{i}^{4}\beta_{\mathrm{ii}}X_{i}^{2}+\sum \sum_{i<j}\beta_{\mathrm{ij}}X_{\mathrm{ij}}$$

where Y represents the dependent variable, $\beta_{0}$, $\beta_{i}$ and $\beta_{\mathrm{ii}}$are linear coefficient, quadratic coefficient and interaction coefficients respectively, need to be estimated, and $X_{i}$ represents the independent variables.

Thirty runs are required for FCCD to cover all possible combination of X_1, X_2, X_3, and X_4 distributed as follows: sixteen runs for the factorial design, eight runs are for axial (star) points and six runs at the center of the design [4], [5]. To avoid or minimize the effect of unexpected variability in the responses, thee experiments were run in random order. The data for the thirty-run of FCCD with the coded and actual levels of the four factors are given in Table 3.

### 2.4 Analytical methods

COD, color and NH_3_–N, were immediately tested before and after each experiment. Leachate sample was shacked well analyzed. NH_3_–N concentration was measured by the Phenol Method No. (4500) using a UV–vis spectrophotometer at 640 nm with a light path of 1 cm or greater. pH was measured using a portable digital pH/Mv meter. COD concentration was determined by the open reflux method No. (5220). The test values are presented as the average of the three measurements, and the difference between the measurements of each value was less than 3%. The removal efficiencies of COD and NH_3_–N were obtained using the following Eq. (5):

(5) Removal(%)=[(Ci–Cf)/Ci]×100

where *C_i_* and *C_f_* refer to the initial and final COD and NH_3_–N concentrations respectively.
