# Supplementary material for: Performance of combined persulfate/aluminum sulfate for landfill leachate treatment
Source: Data Brief. 2018 May 24;19:951–8. doi: 10.1016/j.dib.2018.05.111 (PMC5997951; doi:10.1016/j.dib.2018.05.111)
Supplement: Supplementary file 1 — Supplementary material [file mmc1.docx]

**Conflict of interest**

There is no any conflict of interest in this paper.
