# Supplementary material for: Performance of combined persulfate/aluminum sulfate for landfill leachate treatment
Source: Data Brief. 2018 May 24;19:951–8. doi: 10.1016/j.dib.2018.05.111 (PMC5997951; doi:10.1016/j.dib.2018.05.111)
Supplement: Supplementary file 2 — Supplementary material [file mmc2.docx]

Table 4. The results of ANOVA for color, COD and NH3–N removals

| Color removal % (R-square = 0.904) |
| --- |

Sum of Mean F
 Source Squares DF Square Value P-value F

Model 896.92 14 64.07 10.09 < 0.0001

$X_{1}$ 8.83 1 8.83 1.39 0.2565
 $X_{2}$ 154.41 1 154.41 24.32 0.0002
 $X_{3}$ 3.71 1 3.71 0.58 0.4565
 $X_{4}$ 193.45 1 193.45 30.47 < 0.0001
 $X_{1}^{2}$ 0.58 1 0.58 0.091 0.7665
 $X_{2}^{2}$ 245.70 1 245.70 38.71 < 0.0001
 $X_{3}^{2}$ 101.10 1 101.10 15.93 0.0012
 $X_{4}^{2}$ 27.31 1 27.31 4.30 0.0557
 $X_{1}X_{2}$ 9.33 1 9.33 1.47 0.2441
 $X_{1}X_{3}$ 51.84 1 51.84 8.17 0.0120
 $X_{1}X_{4}$ 53.36 1 53.36 8.41 0.0110
 $X_{2}X_{3}$ 113.00 1 113.00 17.80 0.0007
 $X_{2}X_{4}$ 16.61 1 16.61 2.62 0.1266
 $X_{3}X_{4}$ 0.45 1 0.45 0.071 0.7939
 Residual 95.22 15 6.35
 Lack of Fit 67.20 10 6.72 1.20 0.4458
 Pure Error 28.02 5 5.60
 Total 992.14 29

| COD removal % (R-square = 0.901) |
| --- |
| Sum of Mean F   Source Squares DF Square Value P-value  Model 2206.30 14 157.59 9.82 < 0.0001  $X_{1}$ 84.41 1 84.41 5.26 0.0367  $X_{2}$ 1.60 1 1.60 0.100 0.7564  $X_{3}$ 52.87 1 52.87 3.29 0.0896  $X_{4}$ 97.44 1 97.44 6.07 0.0263  $X_{1}^{2}$ 527.70 1 527.70 32.88 < 0.0001  $X_{2}^{2}$ 211.90 1 211.90 13.20 0.0025  $X_{3}^{2}$ 414.18 1 414.18 25.80 0.0001  $X_{4}^{2}$ 1.19 1 1.19 0.074 0.7889  $X_{1}X_{2}$ 266.67 1 266.67 16.61 0.0010  $X_{1}X_{3}$ 169.78 1 169.78 10.58 0.0054  $X_{1}X_{4}$ 153.02 1 153.02 9.53 0.0075  $X_{2}X_{3}$ 17.89 1 17.89 1.11 0.3078  $X_{2}X_{4}$ 5.62 1 5.62 0.35 0.5630  $X_{3}X_{4}$ 124.77 1 124.77 7.77 0.0138  Residual 240.77 15 16.05  Lack of Fit 204.48 10 20.45 2.82 0.1322  Pure Error 36.28 5 7.26  Total 2447.07 29   \| NH3-N removal % (R-square = 0.86) \| \| --- \|   Sum of Mean F   Source Squares DF Square Value P-value  Model 1557.91 14 111.28 6.70 0.0004  $X_{1}$ 1.92 1 1.92 0.12 0.7385  $X_{2}$ 19.41 1 19.41 1.17 0.2967  $X_{3}$ 142.58 1 142.58 8.59 0.0103  $X_{4}$ 276.75 1 276.75 16.67 0.0010  $X_{1}^{2}$ 186.08 1 186.08 11.21 0.0044  $X_{2}^{2}$ 86.57 1 86.57 5.21 0.0374  $X_{3}^{2}$ 13.77 1 13.77 0.83 0.3769  $X_{4}^{2}$ 192.30 1 192.30 11.58 0.0039  $X_{1}X_{2}$ 41.86 1 41.86 2.52 0.1332  $X_{1}X_{3}$ 52.27 1 52.27 3.15 0.0963  $X_{1}X_{4}$ 117.29 1 117.29 7.06 0.0179  $X_{2}X_{3}$ 34.22 1 34.22 2.06 0.1716  $X_{2}X_{4}$ 22.28 1 22.28 1.34 0.2648  $X_{3}X_{4}$ 127.92 1 127.92 7.70 0.0141  Residual 249.07 15 16.60  Lack of Fit 215.81 10 21.58 3.24 0.1030  Pure Error 33.26 5 6.65  Total 1806.98 29 |
